# Prenatal maternal psychosocial stress and risk of asthma and allergy in their offspring: protocol for a systematic review and meta-analysis

**DOI:** 10.1038/npjpcrm.2016.21

**Published:** 2016-05-19

**Authors:** Catherine Flanigan, Aziz Sheikh, Bright I Nwaru

**Affiliations:** 1 Asthma UK Centre for Applied Research, Centre for Medical Informatics, Usher Institute of Population Health Sciences and Informatics, The University of Edinburgh, Edinburgh, UK; 2 School of Health Sciences, University of Tampere, Tampere, Finland

## Background

Asthma and allergic disorders are of global concern, with asthma estimated to affect 334 million people and 14% of children worldwide.^1,2^ Although the prevalence of asthma may have plateaued at ~8–12% in some economically developed countries, the global burden remains substantial.^1,3,4^ Although the prevalence of allergic disorders in childhood, such as atopic eczema and allergic rhinitis, varies substantially,^5,6^ the prevalence of atopic eczema appears to be increasing in Europe, Asia and Africa.^7^

In addition to genetic predisposition,^8^ urbanisation,^2^ reduced gastrointestinal flora biodiversity in infancy,^9^ early-life exposure to cigarette smoke,^10^ allergens^11^ and reduced exposure to respiratory infections^12^ are established risk factors for the development of asthma and allergy in childhood. A recent randomised controlled clinical trial has also demonstrated early introduction of peanuts into infants’ diets to be protective against subsequent peanut allergy.^13^ However, many factors remain unclear, and discerning causal pathways and potential targets for intervention is part of the World Health Organization’s strategy for the prevention and control of asthma and allergy.^2^

There is evidence that the susceptibility for developing asthma and allergy is established *in utero*.^14–17^ Certain prenatal exposures are known to influence fetal development, including alcohol, smoking, illicit drugs and teratogenic medications.^18^ The concept of fetal programming describes how intrauterine factors can have an impact on subsequent offspring’s physiology and development,^19^ affecting the risk of chronic diseases later in life.^18^ Childhood asthma and allergy are thought to be similarly influenced,^20^ as adaptive immunity develops prenatally with allergen-specific immune responses demonstrable in newborns.^15,16,21^ Umbilical cord blood has been shown to contain fetally derived immunoglobulin E.^14,16^

Prenatal psychosocial stress, mediated through increased activity of the hypothalamic–pituitary axis, has been postulated as an intrauterine exposure that may influence asthma and allergy susceptibility in the offspring.^12^ The hypothalamic–pituitary axis is a biochemical pathway in which hormones secreted by the hypothalamus and pituitary gland in the brain stimulate the release of cortisol, adrenaline and noradrenaline from the adrenal glands in the abdomen.^22^ During pregnancy, these substances can be transmitted to the fetus and influence its development.^18,23^ Maternal prenatal psychosocial stress increases the risk of prematurity,^18^ low birthweight,^18^ offspring neurodevelopmental and cognitive delay, attention-deficit hyperactivity disorder, and other mental health disorders in both animals and humans.^18,19,22,23–25^ Maternal prenatal psychosocial stress may also cause epigenetic effects with DNA methylation and altered gene expression in the placenta, although the significance of this has yet to be determined.^25^ In addition, there is some evidence that prenatal stress exposure can influence the composition of offspring’s intestinal microbiota and also result in increased susceptibility to asthma.^26^

Animal studies have demonstrated that high levels of cortisol generated by prenatal psychosocial stress can increase airway responsiveness in the offspring^27–29^ and potentiate cell differentiation from T-helper cell-type 1 (Th1) to Th2 phenotype. Epidemiological studies have now investigated the potential impact of different indicators of maternal psychosocial stress during pregnancy, including bereavement,^30–32^ exposure to natural disasters,^33^ anxiety and depression symptoms,^34,35^ on the risk of asthma and allergy in the offspring. However, to better appreciate the underlying evidence base on the role of psychosocial stress in the development of asthma and allergy in the offspring, it is important to undertake a synthesis of primary studies that have emerged on this topic. This will provide the opportunity to identify key indicators of psychosocial stress that may influence the risk of asthma and allergy in children and the effects of which may be modified through development of evidence-based and tailored primary prevention interventions.

## Aims

The aim of the study is to identify, critically appraise and synthesise primary studies investigating the role of maternal prenatal psychosocial stress and adverse life events in the development of asthma and allergy in the offspring.

## Methods

This protocol has been developed in line with the Preferred Reporting Items for Systematic reviews and Meta-analysis Protocols (PRISMA-P) guidance^36^ and draws on the Cochrane Public Health Group’s guideline for developing a systematic review protocol.^37^

### Study types

The following study types will be considered for inclusion: experimental studies (i.e., randomised controlled trials, quasi-randomised controlled trials, controlled clinical trials, controlled before-and-after studies and interrupted time-series designs); both retrospective and prospective cohort studies; case–control studies; and cross-sectional studies. Reviews, case studies, case series and animal studies will be excluded.

### Participants

Participants will include pregnant women and their offspring of any age.

### Exposure

There is no single definition of psychosocial stress; for the purposes of this study, it is an encompassing term describing any element of our social environment or individual emotional state that places greater demands on us than we can easily adjust to.^38^ To reflect the diversity of factors that constitute psychosocial stress, all indicators of acute or chronic stressors or negative life events are eligible for inclusion. From a scoping literature search, the following examples of psychosocial stress are anticipated: depression or anxiety disorder, pregnancy-related anxiety,^24^ problems with pregnancy,^30^ poor maternal–fetal attachment,^34^ issues with existing children,^30^ exposure to violence, discrimination or prejudice,^39^ financial problems, residential move^30^ or housing issues,^39^ daily stressors, or generalised psychological stress or distress.^35^ Sources of adverse life events include bereavement, natural disasters,^33^ separation, divorce or marital problems, involuntary job loss for mother or partner and homelessness.

### Outcomes

Primary outcomes will include any allergic disorder as defined by the World Allergy Organisation in their recommendations in nomenclature; this includes asthma, as over 80% of cases are allergic in aetiology.^40^ The following are the primary outcomes: asthma, atopic dermatitis/eczema, atopic sensitisation, food allergy, allergic rhinitis, urticaria and anaphylaxis.

All primary outcomes, with the exception of atopic sensitisation, are defined either by physician assessment within the study or by the self-report of a physician diagnosis. This reflects the use of clinical diagnosis as the primary method of diagnosis of allergic conditions. In addition, asthma diagnosis through the use of airway function tests including peak expiratory flow, FEV_1_ (forced expiratory volume in 1 s), forced vital capacity, forced expiratory flow rate or alternative age-appropriate pulmonary function tests (oscillometry or exhaled nitric oxide analysis) are also accepted methods of assessment.^33^

Atopic sensitisation is a physiological diagnosis and is defined either by clinical assessment of skin-prick testing or measurement of raised antigen-specific immunoglobulin E. Diagnosis of food allergy or urticaria through clinical assessment of skin patch test or similar method is also an accepted outcome.

Secondary outcomes will include any measure of disease severity or impact, including frequency of asthma exacerbations, use of asthma medications, hospitalisation for asthma, wheezing as defined by self-report or physician diagnosis^34^ and measures of health-related quality of life.

### Exclusion criteria

Exclusion criteria are as follows: animal studies, studies in which the exposure was physical stress including obesity, studies in which low socioeconomic status alone is the exposure of interest and studies that do not clearly distinguish between prenatal and postnatal stress.

### Study identification

The following databases will be searched for relevant studies from 1960 to the present day: MEDLINE, EMBASE, Cochrane Library, Web of Science, Scopus, Global Health and Cab International; World Health Organization global library; PsychINFO, CINAHL, AMED, National Health Service Evidence Health Information Resources and Google Scholar. The following databases for international conference proceedings will also be searched: Conference Proceedings Citation Index via Web of Knowledge and Zetoc via British Library. Reference lists of eligible articles will be hand-checked for additional citations. International experts in the field will be contacted to ask for any relevant studies not ascertained by our searches. We will also search the grey literature through Open Grey and The Grey Literature Report. Finally, the following registers will be searched to locate ongoing studies: The Cochrane Central Register of Controlled Trials, International Standardized Randomised Control Trial Number Registry, World Health Organization International Clinical Trials Registry Platform, ClinicalTrials.gov, Australian and New Zealand Clinic Trials Registry, and Current Controlled Trials. Search strategies have been developed for all intended database searches and are included in Appendix 1.

### Study selection

The records retrieved from the databases will be exported to Endnote Library for study screening, de-duplication and overall management of the retrieved records. All study titles and abstracts resulting from the above searches will be independently screened by two reviewers in respect to the inclusion and exclusion criteria of the review; a third reviewer will arbitrate any disagreements that are not resolved by discussion. The same process will be repeated for full-text screening. There will be no language restriction, and where possible efforts will be made to translate studies found in any language other than English. Multiple reports utilising one data set or analysed from the same study will be reported as one study. Where data or information are missing, every effort will be made to contact the authors requesting additional information.

### Data extraction and management

A standardised form has been developed for data extraction (Appendix 2). The data extraction form will be piloted and revised accordingly before use in the review. Data extraction will be completed independently by two reviewers and discrepancies will be resolved by referral to a third reviewer for arbitration.

### Quality assessment

All studies identified will be assessed for their quality and potential for risk of bias by two independent reviewers. Quality and risk of bias in observational studies will be evaluated using the Effective Public Health Practice Projec tool,^41^ whereas we will use the Cochrane Effectiveness and Practice Organization of Care guidelines for experimental studies.^37^ The Effective Public Health Practice Project tool was selected, as in addition to a global rating of study quality it also provides individual ratings for six domains of study quality assessment enabling more detailed assessment of strengths and weakness of individual studies. The intention is that the quality grading of each of the six domains will then inform an overall grading for each study. For each study, the grading for the domains individually and the global study rating will be assigned categories of risk of bias: low, moderate and high. Any disputes not resolved by discussion will be arbitrated by a third reviewer.

### Analysis

All included studies will be descriptively presented in a tabular form, summarising key features of the study design, exposure types and methods of assessment, outcomes and methods of assessment and an indicator of risk of bias in each study. We will undertake both narrative synthesis of the evidence and quantitative pooling (meta-analysis) of studies judged largely to be homogeneous with regard to the clinical, methodological and statistical aspects. Fixed-effect or random-effect meta-analyses will be undertaken depending on an assessment of the clinical comparability of studies and assessment of statistical heterogeneity.^42^ We will quantify any heterogeneity between studies using the *I*^2^-test, and where *I*^2^⩾50% we will evaluate possible reasons for observed heterogeneity between studies by undertaking different scenarios of subgroup analyses. In cases in which data are available, we will undertake the following subgroup analyses for each outcome: by type of stress exposure or adverse life events, trimester of pregnancy at the time of exposure, age of child at diagnosis/assessment of outcome (where possible using the following age groups: <5, 5–12 and >12 years) and gender.

Sensitivity analyses will be undertaken by evaluating whether the level of risk of bias in studies has any influence on the pooled risk estimates; other potential sensitivity analyses will be undertaken as possible with the data emanating from the studies. We will use the funnel plot to evaluate the potential of publication bias, and the Trim and Fill approach to explore possible influence of publication bias on the results.^37^ Statistical analysis will be undertaken using Stata Statistical Software (Release 13, StataCorp LP, College Station, TX, USA).

### Grading of the overall strength of the evidence

We will evaluate the strength and quality of the overall evidence using the GRADE (Grading of Recommendations Assessment, Development and Evaluation) approach.^43,44^ As recommended by the GRADE system, in the first stage, we will rate each outcome on a scale of 1–9: scale 7–9 represents outcomes that are critical for clinical decision-making; 4–6 for outcomes that are important but not critical for clinical decision-making; and 1–4 for outcomes that are not important for clinical decision-making and of lower importance to patients. Each outcome will be grouped according to each of these categories.^43,44^ In the second stage, we will appraise the quality of the overall evidence for each outcome, which will be presented using the GRADE evidence profiling table template.

### Registration and reporting

This study has been registered with the University of York Centre for Reviews and Dissemination International prospective register of systematic reviews (PROSPERO), registration number CRD42016036456. The review will be reported in accordance with the PRISMA guidelines for reporting of systematic reviews and MOOSE guidelines for meta-analysis of observational epidemiological studies.^36,45^ Any amendments to the protocol and rationale for such modifications during the systematic review will be reported in the final report.

### The review team

Study screening, data extraction and quality assessment will be performed independently by CF and BIN. CF has previous experience of undertaking a supervised systematic review. BIN is an epidemiologist working in the field of asthma and allergy and has substantial experience in undertaking systematic reviews. AS and CA will arbitrate any disagreements in the review process and will provide field expertise in synthesising the review. They are both asthma and allergy investigators, research methodologists and have a substantial experience in undertaking systematic reviews.

## Discussion

The emerging evidence, which now demonstrates that increased levels of cortisol, resulting from prenatal psychosocial stress, can increase airway responsiveness in the offspring^26–28^ and consequent increased risk of allergic disorders in the offspring now needs to be synthesised. This will provide a clearer picture of the underlying evidence, help to identify key indicators of psychosocial stress during pregnancy that may influence the susceptibility of developing asthma and allergy in children and indicate better pathways in tailoring potential primary prevention interventions.

## Relevance to other reviews

Three related systematic reviews have been recently published on the topic. Exley *et al.* synthesised the association between early childhood adverse events and subsequent onset of asthma in childhood. Hence, by considering early childhood adverse events, their review does not address the specific question of the current review: the role of maternal prenatal psychosocial stress and adverse life events in the development of asthma and allergy in the offspring.^46^ The study by Tibosch *et al.*^47^ did not specifically focus on prenatal maternal stress, but considered the impact of both the child’s psychosocial stress and that of a caregiver (including studies on maternal prenatal stress, both parental stress and other caregivers of the child). For studies looking at maternal stress, the authors included only maternal mental health issues, depression and anxiety, and not broader sources of chronic stress, such as work-related stress or financial difficulties. Furthermore, the authors included only longitudinal studies, considered asthma as the only outcome and searched only three databases (Medline, Pubmed and PsychINFO) for their review, giving a possibility that several studies not indexed in these databases might have been missed. In our current review, by taking a more comprehensive literature search (i.e., including the leading 13 electronic databases and supplemented by contacting experts in the field), we seek to elucidate the role of maternal prenatal stress, independent of maternal postnatal stress and the child’s early-life stress, in the development of asthma, as well as other allergic outcomes in the offspring. In addition, our review intends to encompass both mental health problems and also other broader sources of psychosocial stress that are commonly encountered during pregnancy. Considering that no clinical trial has so far been undertaken on the topic, we will include studies with both retrospective and prospective designs, and where possible undertake sensitivity analysis to evaluate whether evidence emanating from both study design types varies.

With regard to the last and most recent review, the authors synthesised the evidence on the impact of maternal prenatal psychosocial stress on the onset of asthma and wheezing (as the only outcomes) in the offspring.^48^ By the time we were planning the current review, no protocol was published for that review, nor was it registered in PROSPERO; hence, our preliminary search did not pick it up. Nevertheless, by limiting the outcomes to only asthma and wheezing and excluding other allergic outcomes, we believe that there remains a research gap in this evidence base. The impact of maternal prenatal stress on outcomes other than asthma, such as atopic eczema, allergic rhinitis, lung function performance and food allergies, is a key clinical and public health question. By taking a more comprehensive approach (i.e., including the wide range of allergic outcomes) in synthesising the underlying evidence, the current review provides an opportunity to compare the influence of maternal predisposition with psychosocial stress during pregnancy on asthma and the different allergic outcomes in the offspring. Therefore, by including asthma and other allergic outcomes, we plan to use the GRADE approach in appraising the overall evidence and grade the quality of the evidence for each outcome, thus providing a clearer picture of the impact of maternal prenatal psychosocial stress on asthma and specific allergic outcomes in the offspring. Overall, through the current synthesis, we aim to identify key indicators of prenatal psychosocial stress that have a greater impact on offspring’s disease risk that can be amenable to primary prevention interventions; identify potential critical window of impact; identify and grade the evidence on the asthma and allergy outcomes that may be of greater clinical and public concern; and highlight existing gaps in the evidence base and suggestions for future research.

## Conclusions

Asthma and allergic conditions are common chronic disorders with significant health and economic costs for the individual and for the society. Several environmental and lifestyle risk factors have already been identified, and the role of maternal prenatal stress and adverse life events in disease risk in the offspring is currently gaining attention. By undertaking a comprehensive synthesis of primary studies that have now emerged on this topic, we can begin to better appreciate the import and quality of the overall evidence base, thereby allowing us to identify factors that may be amenable to primary prevention interventions.

## Appendix 1: Search Strategies Developed for Databases

**MEDLINE**

1) Stress, Psychological/

2) exp Stress Disorders, Traumatic/ or exp Stress Disorders, Post-Traumatic/ or exp Stress Disorders, Traumatic, Acute/

3) stress*.mp.

4) exp Anxiety/ or exp Anxiety Disorders/ or anxi*.mp.

5) exp Depression/

6) depress*.mp.

7) exp Life Change Events/ or negative life events.mp.

8) adverse life events.mp.

9) natural disasters.mp. or exp Disasters/

10) exp Death

11) exp Bereavement/ or bereave*.mp

12) exp Widowhood

13) 1 or 2 or 3 or 4 or 5 or 6 or 7 or 8 or 9 or 10 or 12

14) exp Pregnancy/

15) pregnan*.mp

16) antenatal.mp.

17) prenatal.mp.

18) exp Prenatal Care/

19) 14 or 15 or 16 or 17 or 18

20) asthma*.mp. or exp Asthma/

21) wheez*.mp.

22) exp Respiratory Hypersensitivity/

23) exp Hypersensitivity, Immediate/ or exp Hypersensitivity/ or exp Hypersensitivity, Delayed/

24) atop*.mp.

25) allerg*.mp. or exp ‘Allergy and Immunology’/

26) exp Dermatitis, Atopic/ or dermatitis.mp.

27) exp Eczema/ or eczema.mp.

28) exp Rhinitis, Allergic/ or exp Rhinitis, Allergic, Seasonal/ or exp Rhinitis, Allergic,Perennial/

29) exp Conjunctivitis, Allergic/

30) hayfever.mp.

31) hay fever.mp

32) rhinoconjuncivitis.mp.

33) food allerg*.mp. or exp Food Hypersensitivity/

34) exp Anaphylaxis/ or anaphyla*.mp.

35) 20 or 21 or 22 or 23 or 24 or 25 or 26 or 27 or 28 or 29 or 30 or 31 or 32 or 33 or 34

36) 13 and 19 and 35

**EMBASE**

1. exp stress/

2. exp prenatal stress/ or exp acute stress disorder/

3. stress/ or stress.mp.

4. exp anxiety/ or exp ‘mixed anxiety and depression’/ or exp anxiety disorder/ or exp generalized anxiety disorder/

5. anxi*.mp.

6. exp depression/

7. depress*.mp.

8. Life change events.mp. or exp life event/

9. negative life events.mp.

10. adverse life event.mp.

11. exp disaster/ or natural diaster*.mp.

12. exp death/

13. exp bereavement/ or bereav*.mp.

14. exp widow/

15. 1 or 2 or 3 or 4 or 5 or 6 or 7 or 8 or 9 or 10 or 11 or 12 or 13 or 14

16. exp pregnancy/

17. pregnan*.mp.

18. exp prenatal care/

19. antenatal.mp.

20. prenatal.mp.

21. 16 or 17 or 18 or 19 or 20

22. exp asthma/ or asthma.mp.

23. exp wheezing/ or wheez*.mp.

24. respiratory hypersensitivity.mp.

25. exp immediate type hypersensitivity/ or exp delayed hypersensitivity/ or exp hypersensitivity/

26. exp allergy/ or allerg*.mp.

27. atopy.mp. or exp atopy/

28. exp atopic dermatitis/ or dermatitis.mp.

29. eczema.mp. or exp eczema/

30. exp allergic rhinitis/

31. exp allergic conjunctivitis/

32. exp hay fever/

33. hayfever.mp.

34. exp rhinoconjunctivitis/

35. exp food allergy/ or food allerg*.mp.

36. food hypersensitivity.mp.

37. exp anaphylaxis/ or anaphyla*.mp.

38. urticarial*.mp

39. 22 or 23 or 24 or 25 or 26 or 27 or 28 or 29 or 30 or 31 or 32 or 33 or 34 or 35 or 36 or 37 or 38

39. 15 and 21 and 39

**Cochrane Library**

#1 stress*

#2 MeSH descriptor: [Stress Disorders, Traumatic, Acute] explode all trees

#3 MeSH descriptor: [Stress Disorders, Post-Traumatic] explode all trees

#4 MeSH descriptor: [Stress Disorders, Traumatic] explode all trees

#5 MeSH descriptor: [Stress, Psychological] explode all trees

#6 MeSH descriptor: [Life Change Events] explode all trees

#7 ‘negative life event’

#8 ‘adverse life event’

#9 MeSH descriptor: [Disasters] explode all trees

#10 MeSH descriptor: [Death] explode all trees

#11 MeSH descriptor: [Bereavement] explode all trees

#12 MeSH descriptor: [Widowhood] explode all trees

#13 MeSH descriptor: [Pregnancy] explode all trees

#14 MeSH descriptor: [Prenatal Care] explode all trees

#15 antenatal

#16 prenatal

#17 MeSH descriptor: [Asthma] explode all trees

#18 MeSH descriptor: [Respiratory Sounds] explode all trees

#19 MeSH descriptor: [Respiratory Hypersensitivity] explode all trees

#20 MeSH descriptor: [Hypersensitivity] explode all trees

#21 MeSH descriptor: [Hypersensitivity, Immediate] explode all trees

#22 atop*

#23 MeSH descriptor: [Allergy and Immunology] explode all trees

#24 allerg*

#25 MeSH descriptor: [Dermatitis, Atopic] explode all trees

#26 dermatitis

#27 MeSH descriptor: [Rhinitis, Allergic] explode all trees

#28 MeSH descriptor: [Conjunctivitis, Allergic] explode all trees

#29 hayfever

#30 ‘hay fever’

#31 rhinoconjunctivitis

#32 MeSH descriptor: [Food Hypersensitivity] explode all trees

#33 MeSH descriptor: [Anaphylaxis] explode all trees

#34 MeSH descriptor: [Urticaria] explode all trees

#35 #1 or #2 or #3 or #4 or #5 or #6 or #7 or #8 or #9 or #10 or #11 or #12

#36 #13 or #14 or #15 or #16

#37 #17 or #18 or #19 or #20 or #21 or #22 or #23 or #24 or #25 or #26 or #27 or #28 or #29 or #30 or #31 or #32 or #33 or #34

#38 #35 AND #36 AND 37

**Web of Science**

#40 #39 AND #20 AND#15

#39 #38 OR #37 OR #36 OR #35 OR #34 OR #33 OR #32 OR #31 OR #30 OR #29 OR #28 OR #27 OR #26 OR #25 OR #24 OR #23 OR #22 OR #21

#38 TS=urticari*

#37 TS=anaphyla*

#36 TS=‘food hypersensitivity’

#35 TS=‘food allerg*’

#34 TS=‘hay fever’

#33 TS=hayfever

#32 TS=rhinoconjunctivitis

#31 TS=‘allergic conjunctivitis’

#30 TS=‘allergic rhinitis’

#29 TS=eczema

#28 TS=dermatitis

#27 TS=‘dermatitis, atopic’

#26 TS=allerg*

#25 TS=atop*

#24 TS=hypersensitivity

#23 TS=‘respiratory hypersensitivity’

#22 TS=wheez*

#21 TS=asthma*

#20 #19 OR #18 OR #17 OR #16

#19 TS=‘prenatal care’

#18 TS=prenatal

#17 TS=antenatal

#16 TS=pregnan*

#15 #14 OR #13 OR #12 OR #11 OR #10 OR #9 OR #8 OR #7 OR #6 OR #5 OR #4 OR #3 OR #2 OR #1

#14 TS=widow*

#13 TS=bereav*

#12 TS=death

#11 TS=‘disaster’

#10 TS=‘natural disaster’

#9 TS=‘negative life event’

#8 TS=‘adverse life event’

#7 TS=‘life change event’

#6 TS=depress*

#5 TS=‘anxiety, disorders’

#4 TS=anxi*

#3 TS=‘stress, disorder’

#2 TS=‘stress, psychological’

#1 TS=stress*

**Global Health**

1. exp mental stress/

2. post-traumatic stress disorder.sh.

3. stress*.mp. or exp stress/ or exp stress conditions/

4. anxiety.mp. or exp anxiety/

5. anxi*.mp.

6. exp depression/

7. depress*.mp.

8. life event.mp.

9. adverse life event.mp.

10. negative life event.mp.

11. exp disasters/ or exp natural disasters/

12. exp death/

13. bereavement.mp.

14. exp widows/ or widow*.mp.

15. 1 or 2 or 3 or 4 or 5 or 6 or 7 or 8 or 9 or 10 or 11 or 12 or 13 or 14

16. exp pregnancy/

17. pregnan*.mp.

18. antenatal.mp.

19. prenatal.mp.

20. exp prenatal care/

21. 16 or 17 or 18 or 19 or 20

22. exp bronchial asthma/ or exp asthma/ or asthma*.mp.

23. wheez*.mp.

24. exp respiratory hypersensitivity/

25. exp hypersensitivity/ or exp delayed type hypersensitivity/ or exp immediate hypersensitivity/

26. atop*.mp.

27. allergies.sh. or allerg*.mp.

28. exp atopic dermatitis/ or dermatitis.mp.

29. exp eczema/ or eczema.mp.

30. exp allergic rhinitis/

31. allergic conjunctivitis.mp.

32. hayfever.mp.

33. hay fever.mp.

34. rhinoconjunctivitis.mp.

35. food allergies.sh.

36. food hypersensitivity.mp.

37. exp anaphylaxis/

38. anaphylactoid.mp.

39. urticarial*

40. 22 or 23 or 24 or 25 or 26 or 27 or 28 or 29 or 30 or 31 or 32 or 33 or 34 or 35 or 36 or 37 or 38 or 39

41. 15 and 21 and 40

**WHO Global Library**

(‘Anxiety’ OR ‘Bereavement’ OR ‘Anxiety Disorders’ OR ‘Depressive Disorder’ OR ‘Disasters’ OR stress* OR life event OR widow) AND (pregnan* OR antenatal OR prenatal OR ‘prenatal care’) AND ‘Bronchial Hyperreactivity’ OR ‘Respiratory Hypersensitivity’ OR ‘Asthma’ OR ‘Rhinitis, Allergic’ OR ‘Hypersensitivity’ OR ‘Hypersensitivity, Delayed’ OR ‘Hypersensitivity, Immediate’ OR ‘Conjunctivitis, Allergic’ OR ‘Dermatitis, Atopic’ OR ‘Eczema’ OR food allergy OR anaphyla*)

**PsychINFO**

1. exp Stress/

2. stress*.mp.

3. exp Acute Stress Disorder/ or exp Posttraumatic Stress Disorder/

4. exp Anxiety/

5. anxi*.mp.

6. exp ‘Depression (Emotion)’/

7. depress*.mp.

8. exp Life Experiences/ or exp ‘Experiences (Events)’/

9. exp Life Changes/ or life change events.mp.

10. negative life events.mp.

11. adverse life events.mp.

12.exp Natural Disasters/ or exp Disasters/

13. exp ‘Death and Dying’/

14. bereavment.mp. or exp Bereavement/

15. exp Widows/

16. 1 or 2 or 3 or 4 or 5 or 6 or 7 or 8 or 9 or 10 or 11 or 12 or 13 or 14 or 15

17. exp Pregnancy/

18. exp Prenatal Care/

19. pregnan*.mp.

20. antenatal.mp.

21. prenatal.mp.

22. 17 or 18 or 19 or 20 or 21

23. asthma.mp. or exp Asthma/

24. exp Bronchial Disorders/ or wheezing.mp.

25. hypersensitivity.mp.

26. exp Allergic Disorders/ or allergic.mp.

27. atopy.mp.

28. exp Dermatitis/

29. eczema.mp. or exp Eczema/

30. allergic rhinitis.mp.

31. allergic conjunctivitis.mp.

32. exp Hay Fever/ or hay fever.mp.

33. hayfever.mp.

34. rhinoconjunctivitis.mp.

35. food allergy.mp. or exp Food Allergies/

36. food hypersensitivity.mp.

37. exp Anaphylactic Shock/ or anaphylaxis.mp.

38. anaphylactoid.mp.

39. Urticaria*

40. 23 or 24 or 25 or 26 or 27 or 28 or 29 or 30 or 31 or 32 or 33 or 34 or 35 or 36 or 37 or 38 or 39

41. 16 and 22 and 40

**CINAHL**

S39 S15 AND S21 AND S38

S38 S22 OR S23 OR S24 OR S25 OR S26 OR S27 OR S28 OR S29 OR S30 OR S31 OR S32 OR S33 OR S34 OR S35 OR S36 OR S37

S37 (MH ‘Urticaria+’)

S36 (MH ‘Anaphylaxis’) OR ‘anaphyla*’

S35 (MH ‘Food Hypersensitivity+’) OR ‘food allerg*’

S34 ‘rhinoconjunctivitis’

S33 ""hay fever""

S32 ‘hayfever’

S31 (MH ‘Conjunctivitis, Allergic’)

S30 (MH ‘Rhinitis, Allergic, Perennial’) OR (MH ‘Rhinitis, Allergic, Seasonal’)

S29 (MH ‘Eczema’) OR ‘eczema’

S28 (MH ‘Dermatitis, Atopic’) OR ‘dermatitis’

S27 ‘allerg*’

S26 ‘atop*’

S25 (MH ‘Hypersensitivity+’) OR (MH ‘Hypersensitivity, Immediate+’) OR (MH ‘Hypersensitivity, Delayed+’)

S24 (MH ‘Respiratory Hypersensitivity+’)

S23 ‘wheez*’ OR (MH ‘Respiratory Sounds’)

S22 (MH ‘Asthma+’) OR ‘asthma’

S21 S16 OR S17 OR S18 OR S19 OR S20

S20 ‘antenatal’

S19 ‘prenatal’

S18 (MH ‘Prenatal Care’)

S17 ‘pregnan*’

S16 (MH ‘Pregnancy+’)

S15 S1 OR S2 OR S3 OR S4 OR S5 OR S6 OR S7 OR S8 OR S9 OR S10 OR S11 OR S12 OR S13 OR S14

S14 (MH ‘Widows and Widowers’) OR ‘widow*’

S13 (MH ‘Bereavement+’) OR ‘bereav*’

S12 (MH ‘Death+’)

S11 (MH ‘Natural Disasters’) OR (MH ‘Disasters+’) OR (MH ‘Mass Casualty Incidents’)

S10 ""negative life event""

S9 ""adverse life events""

S8 (MH ‘Life Change Events+’) OR (MH ‘Life Experiences+’)

S7 ‘depress*’

S6 (MH ‘Depression+’)

S5 ‘anxi*’

S4 (MH ‘Anxiety+’) OR (MH ‘Anxiety Disorders’)

S3 ‘stress*’

S2 (MH ‘Stress Disorders, Post-Traumatic+’)

S1 (MH ‘Stress, Psychological+’)

**AMED**

1. exp Stress psychological/

2. exp Stress disorders post traumatic/

3. stress*.mp.

4. exp Anxiety/

5. exp Anxiety disorders/

6. exp Depression/

7. depress*.mp.

8. exp Life change events/

9. negative life event.mp.

10. exp Disasters/

11. natural disaster*.mp.

12. exp Death/

13. exp Bereavement/

14. widow*.mp.

15. 1 or 2 or 3 or 4 or 5 or 6 or 7 or 8 or 9 or 10 or 11 or 12 or 13 or 14

16. exp Pregnancy/

17. pregnan*.mp.

18. prenatal.mp.

19. antenatal.mp.

20. exp Prenatal care/

21. 16 or 17 or 18 or 19 or 20

22. exp Asthma/ or asthma*.mp.

23. wheezing.mp.

24. exp Respiratory hypersensitivity/

25. exp Hypersensitivity/

26. atop*.mp.

27. allergy.mp.

28. exp Dermatitis/

29. exp Eczema/ or eczema.mp.

30. exp Rhinitis/

31. hayfever.mp.

32. exp Hay fever/

33. rhinoconjunctivitis.mp.

34. exp Food hypersensitivity/ or food allergy.mp.

35. exp Anaphylaxis/

36. anaphylactic.mp.

37. urticaria*

38. 22 or 23 or 24 or 25 or 26 or 27 or 28 or 29 or 30 or 31 or 32 or 33 or 34 or 35 or 36 or 37

39. 15 and 21 and 38

## Appendix 2: Data Extraction Form

**General Information**

**Reviewer**

Date of data extraction

Author

Article title

Source (e.g., Journal) Year/ Volume/ Pages/ Country of origin)

Contact email

Study design

Study aim

Quality assessment

**Population characteristics**

**Cohort**

Sources of subjects

Inclusion criteria

Exclusion criteria

Recruitment procedures used

Total number of participants recruited

Total number of participants eligible for study data collection

Total number of participants responded

Total number of mother-child pairs to complete follow up

Characteristics of cohort group

- Age

- Geographical region

- Socio-economic status

- Ethnicity

- High risk (family history)

- Setting

- Others

Methods of follow up

Length of follow up

**Maternal Exposure**

Type of stress/stresses:

- Natural disaster (state which)

- Bereavement

- Work stress

- Divorce or separation

- Financial problem (including involuntary redundancy)

- Exposure to violence

- Anxiety symptoms

- Depression symptoms

- Other

Method of assessment of exposure:

- Method of assessment

- Objective or subjective?

- If questionnaire or scale validated?

Trimester in which exposure measured

- 1st (week 1–13)

- 2nd (week 14–26)

- 3rd (week 27–40+)

- Other

**Outcomes**

For all outcomes state:

- N/A if not included in paper outcomes

Asthma as an outcome:

- Y/N

- Age or ages at which assessed

- Method of assessment

- Objective or subjective?

- If questionnaire or scale validated?

Frequency of asthma exacerbations

Use of asthma medication:

- If yes state which

Hospitalisation for asthma

Airway function test:

- Peak Expiratory Flow (PEF)

- Forced Expiratory Volume in 1 s

- Forced Vital Capacity (FVC)

- Forced Expiratory Flow Rate

- Oscillometry

- Exhaled Nitric Oxide

- Other

Atopic dermatitis/ecezema as an outcome:

- Y/N

- Age of age ranges at which assessed

- Method of assessment

- Objective or subjective?

- If questionnaire or scale validated?

Atopic sensitisation as an outcome:

- Y/N

- Age or age range at which assessed

- Method of assessment

- Objective or subjective?

- If questionnaire or scale validated?

Food allergy:

- Age or age range at which assessed

- Method of assessment

- Objective or subjective?

- If questionnaire or scale validated?

Allergic rhinitis as an outcomes:

- Y/N

- Age or age range at which assessed

- Method of assessment

- Objective or subjective?

- If questionnaire or scale, validated?

Urticaria as an outcome:

- Y/N

- Age or age range at which assessed

- Method of assessment

- Objective or subjective?

If questionnaire or scale, validated?

Anaphylaxis as an outcome:

- Y/N

- Age or age range at which assessed

- Method of assessment

- Objective or subjective?

- If questionnaire or scale, validated?

Primary outcomes:

- No of cases in study population (%)

- No of cases amongst exposed (%)

Estimates of association between maternal stress and primary outcome:

- HR/RR/OR/other

- Crude estimates (95% CI)

- Adjusted estimates

- (95% CI)

- *P*-value

Secondary outcomes:

- No of cases in study population (%)

- No of cases amongst exposed (%)

Estimates of association between maternal stress and secondary outcome(s):

- OR/RR/other

- Crude estimates

- Adjusted estimates

- (95%) CI

- *P*-value

Missing data:

- No lost to follow up and/or incomplete data

- How was it dealt with in analysis

**Analysis**

Apriori power calculation

Method of analysis

Statisical software

Subgroup analysis

Additional analyses results not extracted

Confounders included

Mediators Included

Quality Assessment grade for:

- appropriateness of study design

- selection bias

- outcome assessment

- exposure assessment

- confounder assessment -

- appropriateness of statistical analysis

- overall grading

**Extra useful information**

Limitations

Interpretation

Generalisibility

Funding

Other
